# Protecting newborns from pertussis – the challenge of complete cocooning

**DOI:** 10.1186/1471-2334-14-397

**Published:** 2014-07-17

**Authors:** Pascal Urwyler, Ulrich Heininger

**Affiliations:** 1 Division of Pediatric Infectious Diseases, University Children’s Hospital Basel (UKBB), Spitalstrasse 33, PO Box, CH 4031 Basel, Switzerland

**Keywords:** Pertussis, *Bordetella pertussis*, Vaccine, Cocooning, Neonates

## Abstract

**Background:**

An increase of pertussis cases, especially in young infants and adolescents, has been noted in various countries. Whooping cough is most serious in neonates and young infants in whom it may cause serious complications such as cyanosis, apnoea, pneumonia, encephalopathy and death. To protect newborns and infants too young to be fully immunized, immunization of close contact persons has been proposed (“cocoon strategy”) and implemented in several countries, including Switzerland in 2011. The goal of this study was to assess knowledge about pertussis among parents of newborns and acceptance, practicability and implementation of the recently recommended pertussis cocoon strategy in Switzerland.

**Methods:**

We performed a cross sectional survey among all parents of newborns born between May and September 2012 and 2013 in Basel city and country. Regional statistical offices provided family addresses after approval by the ethical and data protection committees. A standardized questionnaire with detailed instructions was sent to all eligible families. For statistical analyses, independent proportions were compared by Pearson’s chi-squared test.

**Results:**

Of 3546 eligible parents, 884 (25%) participated. All three questions exploring pertussis knowledge were answered correctly by 37% of parents; 25% gave two correct answers, 22% gave one correct answer and in the remaining 16% no answer was correct. Pertussis immunization as part of cocooning was recommended to 20% and 37% of mothers and 14% and 32% of fathers in the 2012 and 2013 study cohorts, respectively. Principal advisors for cocooning were pediatricians (66%) followed by gynecologists/obstetricians (12%) and general practitioners (5%). When recommended, 64% of mothers and 59% of fathers accepted pertussis immunization. The majority of vaccinations were administered in the perinatal period and within 2 months of the child’s birth. However, cocooning remained incomplete in 93% of families and in most families <50% of close contacts received pertussis vaccination.

**Conclusions:**

Implementation of cocooning for protecting newborns from pertussis is challenging and usually remains incomplete. Pertussis immunization rates among close contacts of newborns need to be improved. Ideally, all healthcare providers involved in family planning, pregnancy and child birth should recommend cocooning. Pertussis immunization of pregnant women is an additional measure for optimal protection of newborns and should be promoted.

## Background

Pertussis imposes serious threats to neonates and young infants, such as cyanosis, apnoea, pneumonia, encephalopathy and death [1,2]. Since an all-time nadir in 1976, an increase in pertussis cases in the United States has been observed, with two recent epidemics in 2010 and 2012 [3]. Similar to the US, many other countries worldwide have experienced a resurgence of pertussis in the recent past [4].

Incidence of pertussis decreased after introduction of pertussis whole cell vaccines in the late 1950s in Switzerland. After the switch to pertussis acellular vaccines in the 1990s, pertussis remained under control until recently an increase in cases was noted [2]. The recommended infant immunization schedule in Switzerland comprises 3 doses at 2, 4 and 6 months of age (2, 3 and 4 months in preterm infants <33 gestational weeks), followed by a booster dose at 15–24 (preterm infants: 12) months of age.

Various possible explanations for the re-emergence of pertussis have been postulated, including increased disease awareness, more sensitive diagnostics, waning of vaccine induced and natural immunity, and genetic changes in circulating *Bordetella pertussis* strains [4,5].

Several studies showed that main sources of infection in newborns and young infants were their close contact persons, mostly those living in the same household [6-8]. As there is no reliable passive immunity against pertussis in newborns, indirect protection by reducing the risk of transmission from close contact persons has been put forward as the so called “cocoon strategy” [9-11]. In accordance with the concept of cocooning, national immunization guidelines in Switzerland were adapted twice in the recent past. Firstly, in December 2011, one pertussis booster dose for young adults (25 to 29 years of age) and for all adolescents and adults regardless of age if in contact with young infants (<6 months) was recommended [12]. Secondly, in February 2013, a pertussis booster dose for all adolescents between 11 and 15 years of age and for all women (unless immunized against pertussis within the previous five years) in the second or third trimester of pregnancy was recommended [13]. Despite debates on the cost-effectiveness of the cocoon strategy [14-18] and recent studies performed in animal models, which put into question the ability of acellular pertussis vaccines to prevent transmission of *B. pertussis*[19,20], the cocooning strategy and immunization of pregnant women remain the only implemented strategies specifically aimed at protecting newborns from pertussis in Switzerland. The goal of this study was to assess parental acceptance, practicability and implementation of the recently recommended pertussis cocoon strategy.

## Methods

This cross-sectional survey was designed to investigate knowledge about pertussis as well as attitudes towards and acceptance of pertussis immunization among parents of newborn children. In addition, information about pertussis vaccination rates among siblings and other close contacts of newborns was obtained to determine the completeness of each newborn’s individual cocoon.

### Study population

Parents of all children born between May 1 and September 30, 2012 and 2013, with residency in either the canton of Basel city or Basel country, were eligible for study participation. May 2012 was chosen as the first birth cohort, because the cocoon strategy had been introduced in December 2011 and we estimated that a period of 6 months would be necessary for its implementation. The restriction to the birth cohort of September was due to logistic reasons, as the birth statistics for the 4^th^ quarter of 2013 were not available at the time the surveillance was performed. We therefore decided to test two identical time periods in calendar years 2012 and 2013, i.e. May to September.

Accordingly, addresses of parent(s) of these children were obtained from the respective cantonal statistical offices after approval by the ethical and data protection committees. Parents received a standardized questionnaire together with a cover letter and detailed instructions by mail. They were asked to fill out the questionnaire and send it back by provided postpaid envelopes within 2 weeks. In case of refusal of study participation, we asked them to send back the blank questionnaire. Parents of twins were instructed to send back only one questionnaire.

All correspondence and documents for parents were in German, the main language spoken in Basel. Assistance for study related questions was offered by e-mail and via a telephone hotline.

### Study questionnaire

The standardized study questionnaire was divided in two sections.

The first section consisted of three questions and multiple choice answers to assess parents’ knowledge about pertussis:

1. How well are newborns protected from pertussis? – a) very well, b) well, c) little, d) don’t know

2. For which age group is pertussis most dangerous? - a) young newborns, b) schoolchildren, c) adults, d) for all the same, e) don’t know

3. What is the most common source of infection for newborns - a) other newborns, b) persons of the same household, c) loose contact persons, d) don’t know.

In the second section, detailed demographic household information and immunization data as well as personal attitudes were requested. These included general information about the newborn, such as birth date, sex, nationality and maternity clinic as well as information on all household members. Parental educational level was classified according to the proposal by the International Standard Classification of Education [21].

### Interpretation of data and statistical analysis

According to official Swiss immunization guidelines, pertussis immunization status for adults was considered up to date if the last dose was administered <10 years ago, except for mothers of children born in 2013, for whom the interval was officially reduced to <5 years in 2013 [13]. Siblings were considered up to date with their pertussis immunization if they had received ≥3 doses (current age 6–23 months), ≥4 doses (current age ≥24 months to 7 years), or ≥5 doses (current age 8–17 years).

Anonymized data from questionnaires that were received within 7 weeks after shipment were entered into a central, electronic database. Data were managed and analyzed using Statistical Package for Social Sciences software (version: IBM SPSS Statistics 22, IBM Switzerland, Zurich, Switzerland). Independent proportions were compared by Pearson’s chi-squared test. P-values <0.05 were considered statistically significant. If not indicated otherwise, values are given as means with median and range in brackets.

As this was an exploratory study without pre-defined hypotheses, no sample size calculation was performed.

### Ethics

Performance of this survey was approved by the ethical committee of the cantons of Basel in November 2013 (EKBB 318/13).

## Results

### Response rate

**Figure 1:**
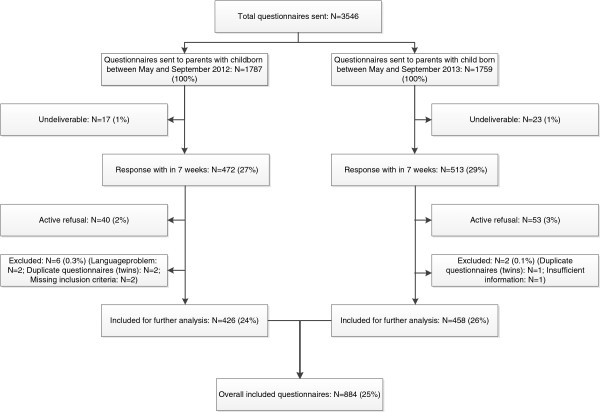
Study flow chart.

A total of 3546 questionnaires were distributed to parents of 1787 children born between May and September 2012 and to parents of 1759 children born between May and September 2013. Response rates were 27% (N = 472) and 29% (N = 513) for the 2012 and 2013 study cohorts, respectively. Altogether 884 (25%) questionnaires were included in the final analyses (Figure 1).

### Study population

General characteristics were very similar for the 2012 and 2013 study cohorts (Table 1). The great majority of infants (81%) were Swiss by nationality and 809 (92%) were born in one of three major maternity hospitals in the region, one of which is a private and two are public hospitals. The size of the newborns’ cocoons was quite variable with a mean of 4 and a range from 1 (single parent) to 11 close contacts (parents, siblings, and other close contact persons).

**Table 1:** General characteristics of study cohorts

	**Birth year 2012**	**Birth year 2013**	**Total**
	**N (%)**	**N (%)**	**N (%)**
**Newborn**	426 (100)	458 (100)	884 (100)
**Sex, known**	426 (100)	457 (99.7)	883 (99.9)
Female	203 (48)	195 (43)	398 (45)
**Nationality, known**	426 (100)	455 (99.3)	881 (99.7)
Swiss^a^	344 (81)	373 (81)	717 (81)
Other	82 (19)	82 (18)	164 (19)
**Maternity clinic, known**	426 (100)	455 (99.3)	881 (99.7)
Hospital A	178 (42)	192 (42)	370 (42)
Hospital B	113 (27)	125 (27)	238 (27)
Hospital C	93 (22)	108 (24)	201 (23)
Other	42 (10)	30 (7)	72 (8)
**Mothers**	426 (100)	458 (100)	884 (100)
**Age (in years), known**	418 (98)	439 (96)	857 (97)
Mean/Median	33/33	33/33	33/33
IQR/Range	30-36/17-46	30-36/20-47	30-36/17-47
**Educational level, known**^**b**^	421 (99)	451 (98)	872 (99)
Compulsory school (ISCED 2)	21 (5)	17 (4)	38 (4)
Apprenticeship (ISCED 3)	130 (31)	147 (32)	277 (31)
Higher education (ISCED 4)	270 (63)	287 (63)	557 (63)
**Fathers**	424 (100)	453 (100)	877 (100)
**Age (in years), known**	402 (95)	412 (91)	816 (93)
Mean/Median	36/35	36/35	36/35
IQR/Range	32-40/21-56	30-36/20-77	32-39/20-77
**Educational level, known**^**b**^	418 (99)	442 (98)	860 (98)
Compulsory school (ISCED 2)	19 (4)	23 (5)	42 (5)
Apprenticeship (ISCED 3)	149 (35)	148 (33)	297 (34)
Higher education (ISCED 4)	250 (59)	271 (60)	521 (59)
**Cocoon size**			
**Siblings**^**c**^	212 (100)	259 (100)	471 (100)
Mean/Range^d^	0.5/0-4	0.57/0-4	0.5/0-4
**Other close contact persons**^**e**^	663 (100)	758 (100)	1421 (100)
Mean/Range^d^	1.6/0-7	1.7/0-5	1.6/0-7
**Total (including parents)**	1725 (100)	1928 (100)	3653 (100)
Mean/Range^d^	4.0/1-11	4.2/1-9	4.1/1-11

^a^Including newborns with multiple citizenships (Swiss plus other).

^b^International Standard Classification of Education Levels.

^c^Twin siblings and subsequently born siblings not included as these were not relevant for cocooning at time of birth of study infant.

^d^Number of close contacts per newborn.

^e^Other than parents or siblings.

### Parental knowledge about pertussis

Results of parental knowledge about pertussis are shown in Table 2. Overall, 50-70% of questions were answered correctly. Higher proportions of questions were answered correctly when mothers filled in the questionnaire as compared to fathers (62% versus 50%; p = 0.002) and more questions were answered correctly in the 2013 cohort compared to the 2012 cohort (64% versus 58%; p = 0.002).

**Table 2:** Parental knowledge about pertussis

**Questionnaire filled in by (N)**	**Birth year 2012**						**Birth year 2013**						**Overall**
	**Mother**	**Father**	**both**	**other**	**unknown**	**Subtotal**	**Mother**	**Father**	**both**	**other**	**Unknown**	**Subtotal**	**Total**
	**301**	**27**	**95**	**1**	**2**	**426**	**310**	**32**	**111**	**1**	**4**	**458**	**884**
**Question 1**^**a**^													
N (%) correct answers	144 (48)	8 (30)	38 (40)	0 (0)	0 (0)	**190 (45)**	164 (53)	11 (34)	71 (64)	0 (0)	4 (100)	**250 (55)**	**440 (50)**
**Question 2**^**b**^													
N (%) correct answers	214 (71)	20 (74)	60 (63)	1 (100)	0 (0)	**295 (69)**	227 (73)	17 (53)	77 (69)	1 (100)	4 (100)	**326 (71)**	**621 (70)**
**Question 3**^**c**^													
N (%) correct answers	189 (63)	15 (56)	52 (55)	1 (100)	1 (50)	**258 (61)**	205 (66)	18 (56)	77 (69)	1 (100)	4 (100)	**305 (67)**	**563 (64)**

^a^How well are newborns protected against pertussis?

^b^For which age group is pertussis most dangerous?

^c^What is the most common source of infection for pertussis in newborn?

Of 884 questionnaires, in 331 (37%) all three questions were answered correctly, 222 (25%) had two correct answers, 193 (22%) had one correct answer and in the remaining 138 (16%) questionnaires none of the three questions was answered correctly.

### Pertussis immunization recommendation and acceptance rates by parents

**Figure 2:**
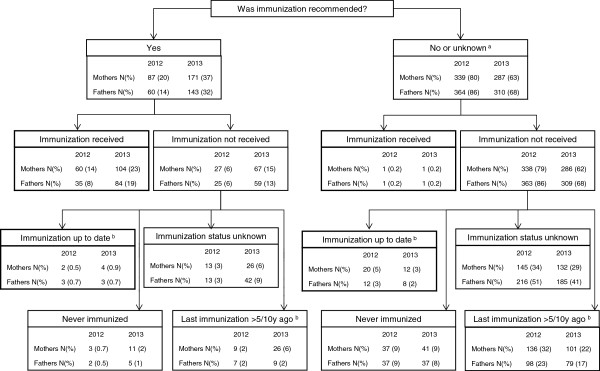
**Pertussis immunization recommendation and immunization status in 1761 parents. **^a^Unknown in 2/1 mothers (2012/2013) and 3/4 fathers (2012/2013). ^b^According to Swiss immunization guidelines defined as last dose <10 years ago, except for mothers, for whom the interval was reduced to <5 years in 2013.

An overview of pertussis immunization recommendations for mothers and fathers of newborns as well as their immunization status is shown in Figure 2.

The proportion of parents who received a pertussis immunization recommendation to indirectly protect their newborns increased from 2012 to 2013: rates were 20% and 37%, respectively, for mothers and 14% and 32%, respectively, for fathers (both p < 0.001). As can be seen, the proportion of fathers who had received the recommendation was lower than that of mothers in 2012 (p = 0.016) and also in 2013, although less pronounced (p = 0.067).

When recommended, the proportion of acceptance of pertussis immunization was similar among mothers (164 of 258, 64%) and fathers (119 of 203, 59%; p = 0.279). It should be noted that an additional 38 mothers and 26 fathers were found to be up to date with their immunization status according to Swiss immunization guidelines as they had received a pertussis vaccine within 5–10 years before their child’s birth. Finally, 2 mothers and fathers apparently had received pertussis immunization for cocooning despite lack of such a recommendation. This means that overall 204 (23%) of 884 mothers and 147 (17%) of 877 fathers (p < 0.001) were accurately protected against pertussis in terms of the cocoon strategy.

### Timing of parental pertussis immunization, impact of advisor, and demographic factors

**Figure 3:**
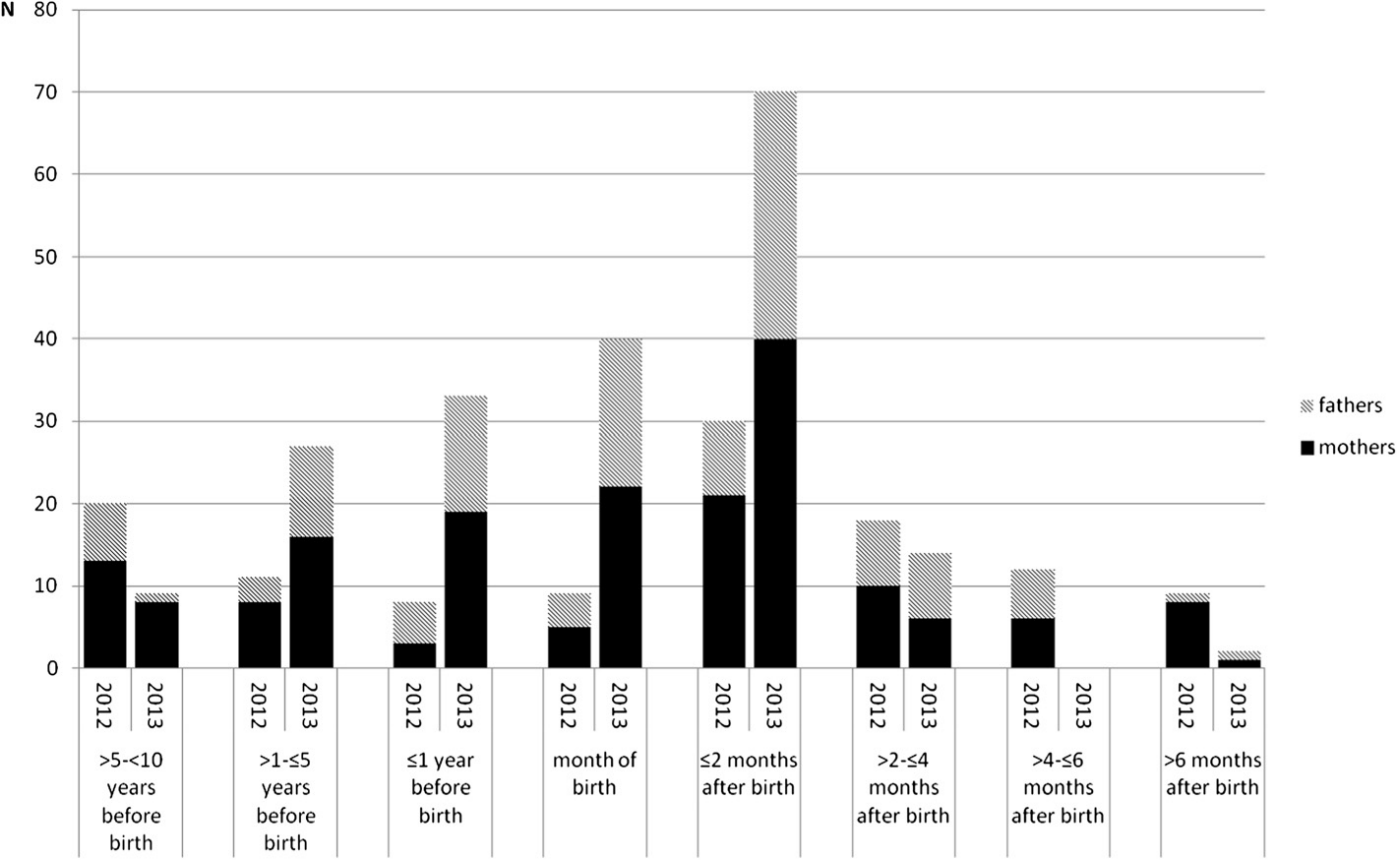
**Time interval between parental pertussis immunization and child birth in 2012 and 2013 study cohorts**^**a**^**. **^a^restricted to immunizations administered <10 years before birth of child; 2012: 74 mothers/43 fathers; 2013: 112 mothers/83 fathers.

When analyzing the date of pertussis vaccine administration for cocooning, a clear peak can be seen during the month of the child’s birth and the two following months with 88 (47%) of 178 immunized mothers and 61 (48%) of 126 immunized fathers vaccinated during the peri- and early postnatal period (Figure 3). In 31 mothers and 24 fathers pertussis immunization was delayed for more than two months after birth of their child.

Further, parental pertussis immunization rates were stratified by time and advisor of the recommendation (Tables 3 and 4). Overall, principal advisors were healthcare professionals, mostly pediatricians (66%) followed by gynecologists/obstetricians (12%) and general practitioners (5%). Pediatricians were most frequently reported as their advisors by parents who received their pertussis immunization in the peri- and postnatal period, whereas gynecologists and pediatricians were equally frequent advisors of parents immunized before birth of the child. Immunization acceptance rates were higher with multiple advisors: 19 (90%) of 21 parents with more than one advisor subsequently received pertussis immunization compared to 264 (60%) of 440 parents with a single advisor (p = 0.02).

**Table 3:** Parental pertussis immunization by time relation to child’s birth and by advisor, 2012 and 2013 study cohorts combined

**Advisor**	**<10 years before birth**			**During month of birth**			**<6 months after birth**			**Overall**
	**Mother**	**Father**	**Subtotal**	**Mother**	**Father**	**Subtotal**	**Mother**	**Father**	**Subtotal**	**Total**
	**N**	**N**	**N (%)**	**N**	**N**	**N (%)**	**N**	**N**	**N (%)**	**N (%)**
**Pediatrician**	6	10	16 (10)	18	12	30 (18)	65	52	117 (72)	163 (66)
**Gynecologist/obstetrician**	13	4	17 (57)	3	1	4 (13)	6	3	9 (30)	30 (12)
**General practitioner**	5	1	6 (50)	1	1	2 (17)	2	2	4 (33)	12 (5)
**Travel medicine clinic**	2	5	7 (88)	0	1	1 (13)	0	0	0 (0)	8 (3)
**Spouse/partner**	0	1	1 (25)	0	3	3 (75)	0	0	0 (0)	4 (2)
**Employer**	2	1	3 (75)	0	0	0 (0)	1	0	1 (25)	4 (2)
**Other**^**a**^	4	0	4 (67)	1	0	1 (17)	1	0	1 (17)	6 (2)
**>1 of the above**	2	1	3 (16)	4	3	7 (37)	6	3	9 (47)	19 (8)
**Total**	34	23	57 (23)	27	21	48 (20)	81	60	141 (57)	246 (100)^b^

^a^Recommendation from internet (N = 2)/from media (N = 1), family member (N = 2), midwife (N = 1).

^b^Different Total compared to Figure 3; advisor unknown in 35 mothers and 20 fathers and 11 parents received immunization >6 months after birth of child.

**Table 4:** Parental pertussis immunization rate following recommendation by advisor, 2012 and 2013 study cohorts combined

**Advisor**^**a**^	**Mothers**	**Fathers**	**Overall**
	**N immunization received/N immunization recommended (%)**	**N immunization received/N immunization recommended (%)**	**N immunization received/N immunization recommended (%)**
**Pediatrician**	99/162 (61)	77/140 (55)	176/302 (58)
**Gynecologist/obstetrician**	22/34 (65)	12/19 (63)	34/53 (64)
**GP**	10/12 (83)	4/7 (57)	14/19 (74)
**Travel medicine clinic**	2/4 (50)	6/8 (75)	8/12 (67)
**Midwife**	5/9 (56)	2/2 (100)	7/11 (64)
**Partner**	0/0 (0)	7/9 (78)	7/9 (78)
**Employer**	5/6 (83)	1/1 (100)	6/7 (86)
**Internet**	4/5 (80)	1/1 (100)	5/6 (83)
**Unknown**	1/3 (33)	1/3 (33)	2/6 (33)
**Other**^**b**^	4/10 (40)	1/5 (20)	5/15 (33)
**>1 of the above**	12/13 (92)	7/8 (88)	19/21 (90)
**Total**	164/258 (64)	119/203 (59)	283/461 (61)

^a^Mentioned if N total > 5.

^b^Friend (N = 5), children’s hospital (N = 4), family (N = 3), recommendation from media (N = 2) or general health promotion (N = 1).

Fathers and mothers of children born in Hospital A had slightly higher recommendation rates in 2012 (18% of fathers and 26% of mothers) than those in Hospitals B and C (13% and 17% and 14% and 20%, respectively). In the 2013 study cohort, recommendation rates were similar in all 3 hospitals.

Nationality of children and parental educational levels had no influence on recommendation or immunization rates of parents (data not shown).

### Reasons for lack of parental pertussis immunization despite recommendation

Of 178 parents who did not receive a pertussis immunization despite recommendation, 171 (96%) indicated at least one reason for lack of immunization. Most frequent reasons were “missed opportunity” (N = 64; 36%), “recommendation was forgotten” (N = 46; 26%), and “fear of perceived side effects” (N = 22; 12%). For a complete list of reasons for decline of pertussis immunization among parents see Table 5*.*

**Table 5:** Parental reasons for lack of pertussis immunization despite recommendation, 2012 and 2013 study cohorts

**Parents not receiving immunization despite recommendation (N)**^**a**^	**Birth year 2012**			**Birth year 2013**			**Overall**
	**Mothers**	**Fathers**	**Subtotal**	**Mothers**	**Fathers**	**Subtotal**	**Total**
	**(27)**	**(25)**	**(52)**	**(67)**	**(59)**	**(126)**	**(178)**
	**N (%)**	**N (%)**	**N (%)**	**N (%)**	**N (%)**	**N (%)**	**N (%)**
**Solicited reasons**^**b**^							
No opportunity	12 (44)	6 (24)	18 (35)	24 (36)	22 (37)	46 (37)	64 (36)
Forgotten	7 (26)	10 (40)	17 (33)	14 (21)	15 (25)	29 (23)	46 (26)
Perceived side effects	4 (15)	1 (4)	5 (10)	11 (16)	6 (10)	17 (13)	22 (12)
Considered not important	2 (7)	4 (16)	6 (12)	5 (7)	8 (14)	13 (10)	19 (11)
Doubts about effectiveness	2 (7)	1 (4)	3 (6)	6 (9)	6 (10)	12 (10)	15 (8)
Cost	2 (7)	1 (4)	3 (6)	1 (1)	1 (2)	2 (2)	5 (3)
Religious beliefs	0 (0)	0 (0)	0 (0)	0 (0)	0 (0)	0 (0)	0 (0)
**Unsolicited reasons**^**b**^							
Believed to be up to date^c^	4 (15)	2 (8)	6 (12)	9 (13)	4 (7)	13 (10)	19 (11)
Recommendation came too late	0 (0)	0 (0)	0 (0)	5 (7)	3 (5)	8 (6)	8 (4)
Recommendation not known	3 (11)	0 (0)	3 (6)	1 (1)	2 (3)	3 (2)	6 (3)
Immunization discouraged by physician	1 (4)	1 (4)	2 (4)	3 (4)	1 (2)	4 (3)	6 (3)
Immunization considered unnecessary	1 (4)	1 (4)	2 (4)	2 (3)	1 (2)	3 (2)	5 (3)
Sceptical about immunizations	0 (0)	0 (0)	0 (0)	1 (1)	2 (3)	3 (2)	3 (2)
Recent tetanus immunization	0 (0)	0 (0)	0 (0)	4 (6)	1 (2)	5 (4)	5 (3)
Risk of pertussis considered to be low	0 (0)	0 (0)	0 (0)	3 (4)	1 (2)	4 (3)	4 (2)
Positive history of pertussis disease	0 (0)	1 (4)	1 (2)	1 (1)	1 (2)	2 (2)	3 (2)
Breastfeeding	2 (7)	N/A	2 (4)	1 (1)	N/A	1 (0.8)	N/A
Other	2 (7)	3 (12)	5 (10)	6 (9)	4 (7)	10 (8)	13 (7)

^a^27/67 mothers (2012/2013) and 24/53 fathers (2012/2013) indicated reasons.

^b^One or more reasons could be given.

^c^Of which 9 parents (6 mothers and 3 fathers) actually were up to date.

### Completeness of cocooning

**Figure 4:**
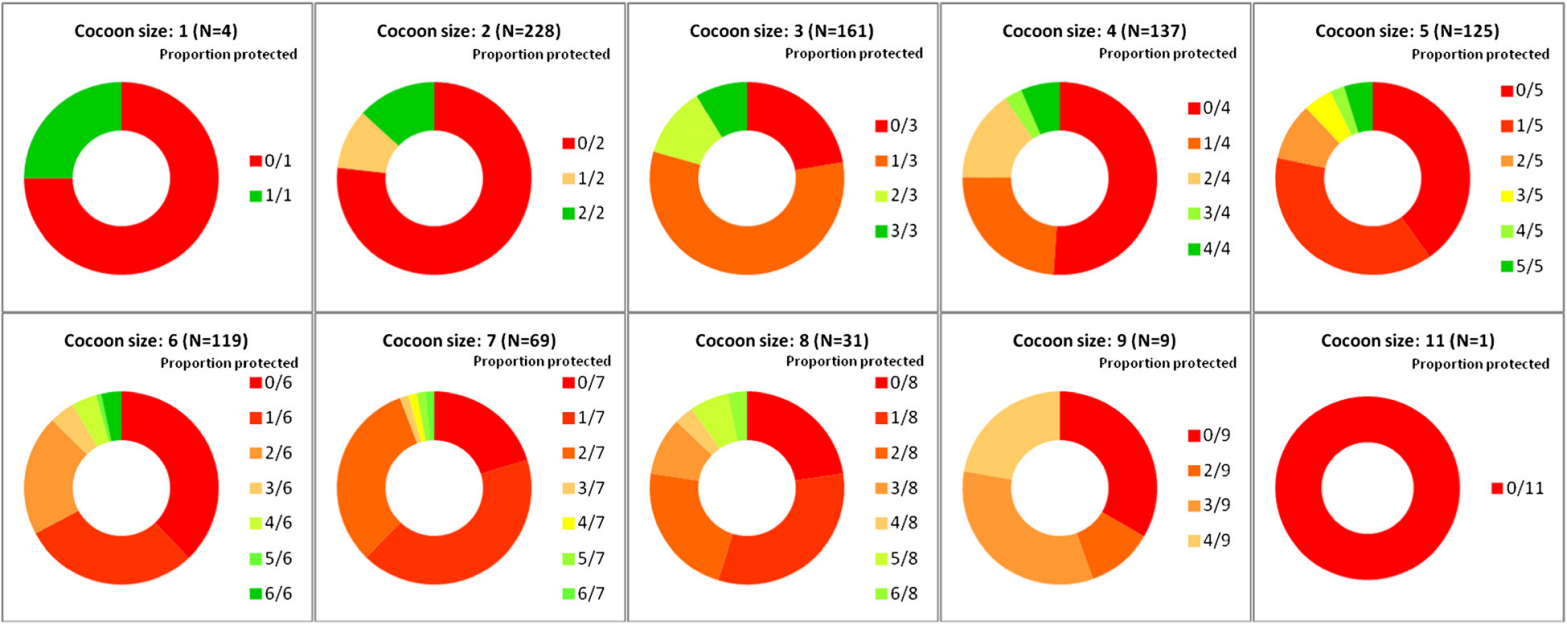
**Completeness of cocooning by cocoon size**^**a **^**in combined 2012 and 2013 study cohorts. **^a^Cocoon size = Number of close contact persons per newborn.

Information provided about other close contact persons of the newborns revealed that immunization status for pertussis was up to date in 79% of siblings, 18% of uncles, 13% of aunts, 7% of grandparents and 1% of day-care providers (Table 6). It should be noted, however, that most parents did not have precise information on the pertussis immunization status of their child’s close contacts not living in the same household, such as aunts and uncles or day care personnel.Each newborn’s individual cocoon was analyzed for completeness of the newborn’s protection by evaluation of the immunization status of all close contact persons combined. This analysis showed that only 64 (7%) of 884 newborns were protected by a complete cocoon (i.e., all close contacts were immunized against pertussis) and merely 158 (18%) newborns were surrounded by a cocoon consisting of ≥50% (indicated by green colored fractions of the cocoons in Figure 4) immunized contact persons.

**Table 6:** Pertussis immunization in close contact persons of newborns – 2012 and 2013 study cohorts

**Contact person**	**Birth year 2012**		**Birth year 2013**		**Total**	
	**N**	**N up to date (%)**	**N total**	**N up to date (%)**	**N total**	**N up to date (%)**
**Siblings**	212	171 (81)	259	201 (78)	471	372 (79)
**Grandmothers**	324	12 (4)	358	38 (11)	682	50 (7)
**Grandfathers**	224	9 (4)	270	29 (11)	494	38 (8)
**Nanny/daycare**	48	0 (0)	30	1 (3)	78	1 (1)
**Aunt**	25	5 (20)	36	3 (8)	61	8 (13)
**Uncle**	16	3 (19)	24	4 (17)	40	7 (18)
**Other**^**a**^	26	0 (0)	40	1 (3)^b^	66	1 (2)

^a^Godmother (N = 17), Godfather (N = 7), Great-grandmother (N = 4), Great-grandfather (N = 1), Great-aunt (N = 2), Friend of family (N = 5), Cousin (N = 7), Niece (N = 1), Other children at daycare (N = 3), Mother’s cousin (N = 1), not specified (N = 18).

^b^Godfather.

## Discussion

### Major findings

This survey uncovered several issues that hinder protection of newborns and young infants from pertussis. Firstly, knowledge among parents of newborns about the threats of pertussis for their child is sparse as is knowledge about the major sources of transmission. Secondly, implementation of the recently introduced concept of cocooning newborns to protect them from pertussis is far from optimal. Similar to experience in the USA, France and Australia, this is mainly explained by healthcare professionals not recommending pertussis immunization to close contact persons of newborns and by knowledge gaps and compliance issues among parents themselves. The fact that “missed opportunities” and “forgotten to get immunized” were the main reasons for not being immunized stated by parents indicates that indirect protection of their infants from pertussis apparently is not high enough on their list of priorities. Accordingly, 19% and 20% of pertussis immunization in mothers and fathers, respectively, were delayed for more than two months after birth of their child, i.e. beyond the time point of the first scheduled pertussis immunization in the child itself.

Immunization of parents before birth of the child, i.e. pregnant women and fathers to be, was less frequently applied than immunization after the birth of the child. With regards to pregnant women this can be explained by the fact that this strategy was only introduced in 2013, whereas with respect to the fathers apparently lack of awareness prevails. In 2012, parents of children born in Hospital A had slightly higher recommendation rates than those in Hospitals B and C but this difference was not discernible any more in 2013. This observation indicates earlier adoption of the new immunization recommendation in Hospital A which was caught up later by Hospitals B and C. Interestingly, pediatricians by far were the most influential group of all healthcare professionals as they provided the great majority of recommendations. However, even if immunization was recommended to parents, their acceptance was only 59% (fathers) and 64% (mothers).

Pertussis immunization was up to date in 79% of siblings of newborns. This figure is disappointing. As siblings were young and therefore in the process of receiving their childhood immunization series, delays or lack of immunization at all should have been addressed and corrected while their mothers were pregnant.

Little is known about the acceptance and modes of communication among other close contact persons as they could not be contacted directly for methodological reasons.

### Comparison to other studies

Two studies conducted in the US [22,23] in a predominantly Hispanic, underinsured, and medically underserved population revealed insufficient knowledge, costs, lack of transportation, and fear of pain/needles as main barriers towards cocooning by immunization. This is different from our setting, i.e. a fairly high educated population with excellent health-care access including reimbursement of recommended immunizations. Here, as mentioned above, actually not having immunization appointments is the major hurdle, whereas fear of side effects plays a minor role (12%) and cost issues play no role at all.

Rates of pertussis immunization following recommendation by healthcare professionals in the range of 61-69% in mothers and 58-59% in fathers in our study population were similar to those achieved by other investigators [10,11]. As one might expect with newly introduced recommendations, rates were higher in 2013 than in 2012 and further increases may be anticipated in the years to come. Whether this assumption is true remains to be shown in further analyses.

### Improving the cocoon strategy towards complete cocooning

Despite considerable increases in the proportion of parents that received a pertussis immunization recommendation in the 2013 study cohort compared to the year before, overall protection remained far too low with only 23% in mothers and 17% of fathers of newborns. Moreover, immunization gaps were also found in siblings and other regular close contact persons. In the latter group, lack of information may have led to an underestimation of the true pertussis immunization rate but in our clinical experience this is unlikely to be a significant proportion. Consequently, 93% of newborns were not surrounded by a complete cocoon protecting them from acquisition of pertussis from close contact persons. We believe that such analysis of completeness of cocooning should be part of counseling during the process of family planning and that all involved healthcare professionals should recommend updating pertussis immunization status of individuals forming the newborn’s cocoon. In this regard, our finding that 90% of parents who received such a recommendation from multiple healthcare professionals actually accepted pertussis immunization is encouraging. Therefore pediatricians, gynecologists and obstetricians, and general practitioners, as well as midwives and nurses, should urgently be sensitized for the concept of cocooning.

Immunization rates of 58-69% following recommendation in our cohort of parents are suboptimal. In contrast, investigators from one center in the US reached an immunization rate of 91% in postpartum women before infant hospital discharge [9]. This impressive success was achieved by using standing orders and providing vaccinations on-site. In the second phase of their study, the investigators expanded their activities to other household members and infant care-givers and reported that at least one other member of the cocoon was immunized in 58% of households. Unfortunately, completeness of cocooning was not reported. Similar to our study, this experience highlights the challenge of providing a complete protective cocoon for the young child. It should be emphasized that providing immunization opportunities, ideally repeatedly, either in hospital or clinic-based settings are likely to improve immunization rates of close contact persons of newborns as the great majority of these individuals are willing to accept pertussis immunization [23].

To improve immunization rates among mothers and potentially protect newborns with maternal pertussis-specific antibodies [24], promotion of immunizing pregnant women on the occasion of pregnancy consultations would be helpful [25-27]. With only 19 mothers (17%) indicating in this study that they were vaccinated against pertussis during the year before giving birth, the concept of pertussis immunization of pregnant women apparently has not been implemented yet and intensified education and promotion is required.

In a Dutch cocooning model, vaccinating siblings provided protection comparable with vaccinating the mother [28]. Therefore, it is advisable for pregnant women who already have children to consult their pediatricians to assure that their children will be up to date with their immunization status when the new child is expected to be born.

At the same time, pregnant women should be encouraged to identify all potential future close contact persons of their child as it has been shown that non-household members with regular contact to the family play a considerable role in pertussis transmission, too [8,28,29].

### Study strengths and limitations

As a strength, this study was surveying predefined parents of newborns irrespective of household composition or any other selection factors besides date of birth and residence. Therefore, the sample can be considered representative. However, since the survey was initiated in German language and voluntary, participation of parents was incomplete. When compared to national data, tertiary education and Swiss nationality were overrepresented among the responders that formed our study cohort [30,31]. This may have introduced a selection bias and therefore the true rate of pertussis immunization among close contacts of newborns is unknown. Language barriers and lack of interest are the most likely explanations for the comparatively low response rate (<30%) in this survey.

## Conclusions

There is an ongoing controversy about cost effectiveness of the cocoon strategy [15-18] and recently published studies in animal models question the ability of acellular pertussis vaccines in preventing transmission of *B. pertussis*[19,20]. Moreover, a recent systematic review of various pertussis immunization strategies failed to identify evidence on the effectiveness of the cocoon strategy [32].

In a recently published Swiss pediatric pertussis surveillance program, the source of transmission remained unknown in 39% of young children hospitalized because of pertussis.^2^ This indicates, that even perfect cocooning will not solve the problem of serious pertussis in young infants. In this regard, neonatal immunization and/or accelerated infant immunization schedules could be promising additional or alternative strategies to protect newborns, but a number of issues about safety and immunogenicity of such immunization schedules need to be solved [4,33-36]. Given that currently available acellular pertussis vaccines have shown to be of limited efficacy both in terms of immediate as well as persistent protection [4], a need for new vaccines such as less reactogenic whole-cell vaccines, better formulations of acellular vaccines, or intranasal live-attenuated vaccines has been identified and some prototypes have undergone preliminary testing [37-39].

Before any of these alternatives may prove to be valid additions to current strategies, we are left with the possibility of better use of currently available vaccines. For the concept of cocooning, this means intensified efforts to improve knowledge about the threats of pertussis in the population and among healthcare professionals, and to optimize pertussis vaccine provision and acceptance by pregnant women and other close contact persons. Although everyone immunized as part of the cocooning strategy will contribute to better control of pertussis in the population beyond his or her individual cocoon, broader protection of the general population by regular booster immunizations throughout life is needed for optimal protection of vulnerable infants.

## Competing interests

PU declares that he has no competing interests. UH is a member of the “Global Pertussis Initiative” and the “Central and Eastern European Pertussis Awareness Group (CEEPAG)” which receive unrestricted educational grants from Sanofi. Further, UH has received lecture fees from all of the manufacturers of acellular pertussis vaccines.

## Authors’ contributions

PU: Acquisition of data, analysis and interpretation of data, first draft of the manuscript. UH: Survey conception and design, supervision of acquisition of data, analysis and interpretation of data, revision of the manuscript, and responsible principal investigator. Both authors read and approved the final manuscript.

## Authors’ information

This work was performed within the framework of PU’s 6 month period of civil service in the division of pediatric infectious diseases and vaccinology, University Children’s Hospital Basel, supervised by UH.

## Pre-publication history

The pre-publication history for this paper can be accessed here:

http://www.biomedcentral.com/1471-2334/14/397/prepub
